# Exergame-Driven High-Intensity Interval Training in Untrained Community Dwelling Older Adults: A Formative One Group Quasi- Experimental Feasibility Trial

**DOI:** 10.3389/fphys.2019.01019

**Published:** 2019-08-07

**Authors:** Stefanie Rebsamen, Ruud H. Knols, Pierrette Baschung Pfister, Eling D. de Bruin

**Affiliations:** ^1^Directorate of Research and Education, Physiotherapy and Occupational Therapy Research Center, University Hospital Zurich, Zurich, Switzerland; ^2^Institute of Physiotherapy, Zurich University of Applied Sciences, Winterthur, Switzerland; ^3^Institute of Physiotherapy, Kantonsspital Winterthur, Winterthur, Switzerland; ^4^Institute of Human Movement Sciences and Sport, Department of Health Sciences and Technology, ETH Zürich, Zurich, Switzerland; ^5^Division of Physiotherapy, Department of Neurobiology, Care Sciences and Society, Karolinska Institute, Stockholm, Sweden

**Keywords:** aerobic exercise, exergaming, feasibility, high-intensity interval training, older adults, virtual reality

## Abstract

**Purpose:**

To investigate the feasibility of an exergame-driven high-intensity interval training (HIIT) and its effects on cardiovascular fitness in untrained community dwelling older adults.

**Methods:**

Twelve older participants [10 women, age 72.3 (*SD*: 4.44) years] performed a high-intensity interval exergame intervention three times a week for 4 weeks. Data was acquired during two baseline and one final measurement. Feasibility outcomes included attrition, adherence, acceptability [Technology Acceptance Model Questionnaire (TAM)], usability [System Usability Scale (SUS)], and enjoyment of exergaming. Furthermore, participants’ physical activity levels pre and post intervention were compared to physical activity levels for similar types of training. The secondary outcome was exercise capacity [heart rate at rest (HRrest), heart rate variability (HRV), maximum heart rate (HRmax), and maximum workload (W, in watt)] evaluated through maximal exercise testing.

**Results:**

Eleven participants completed the study (8% attrition), without any adverse events. Adherence to the HIIT intervention was 91% and participants showed high acceptance of the intervention with TAM scores between 5.8 and 6.7 points. User satisfaction was rated as excellent (SUS total score: 93.5 of 100) and the overall enjoyment of exergaming scored 4.5 of 5 possible points. Total exercise time ranged from 19 to 35 min with a mean of 30.8 (*SD*: 3.6) min. Actual high-intensity exercise time showed consistency with the target exercise time in 98% percent of trainings. Eighty-six percent of high-intensity intervals met the targeted intensity range (>70–90% of HRmax). Thirty-six percent of the recovery periods were completed with a heart rate above the target range of 50–70% of HRmax. Maximum workload (W) during the incremental exercise test post-training increased significantly compared to the baseline measurements one and two (*p* = 0.032, effect size *r* = 0.77 and *p* = 0.012, *r* = 0.87).

**Conclusion:**

High-intensity interval training through exergaming is feasible, safe, and shows high usability and acceptance in community dwelling older people. Exergame-driven HIIT had a significant effect on maximum power output on an incremental exercise test. A more extensive exergame intervention period, higher work to recovery ratios as well as a higher-intensity activity should be considered in future projects.

## Introduction

Physical inactivity is the fourth leading risk factor for mortality globally, accounting for 6% of deaths worldwide and for around 10% in the World Health Organization European Region. Every year, over eight million disability-adjusted life years in the European Region are lost because of insufficient physical activity (Christiansen et al., 2014). Evidence shows major beneficial effects of physical activity on health, such as reducing the risk of most chronic diseases (e.g., cardiovascular disease, obesity, type two diabetes and several cancers), and improving musculoskeletal health as well as psychological well-being (Booth et al., 2002; Christiansen et al., 2014). The evidence for health benefits is overwhelming with risk reductions of at least 20–30% for more than 25 chronic medical conditions and premature mortality (Warburton and Bredin, 2016). Engaging in higher levels of physical activity is, furthermore, associated with higher levels of physical function and independence in older adults (Howes et al., 2017). International physical activity guidelines generally recommend 150 min per week of moderate-intensity or three times 25 min of vigorous-intensity aerobic physical activity per week (Warburton and Bredin, 2016). Even half of this volume of exercise might lead to marked health benefits (Warburton and Bredin, 2016). Despite the evidence on the links between physical activity and health, the majority of Europeans (Europe Commission, 2010) and populations worldwide (Guthold et al., 2018) show insufficient levels of weekly exercise.

In the context of global aging, efforts should be put in optimizing populations’ physiological function (Seals et al., 2016). Maintaining motivation to continue and adhere to exercise is, however, often difficult as participating in conventional exercise may not be appealing enough (Phillips et al., 2004). Establishing optimal ways to encourage older adults to become more physically active in the long term is needed (Bennett and Winters-Stone, 2011). Although it is known that early aerobic exercise training after acute illness would be able to enhance cardiovascular fitness and improve functional recovery (Kim et al., 2013), patients with functional impairment are often not willing or able to adhere to the high-volume low-intensity exercises that are conventionally prescribed (Kessler et al., 2012). Similar patterns are seen in the non-clinical older population (Jefferis et al., 2014; McPhee et al., 2016). Research should, therefore, focus on determining the most effective methods to increase and then maintain physical activity and exercise participation in older adults (Taylor, 2014; Seals et al., 2016).

Several guidelines recommend MCE for habitual exercise training (Haskell et al., 2007; Hallal et al., 2012; Tsukamoto et al., 2016). Similar to MCE, long-term HIIT is a safe exercise intervention (Boutcher, 2011; Cornish et al., 2011; Weston et al., 2014; Ramos et al., 2015) and more effective than (long-term) MCE for increasing exercise capacity as well as metabolic and cardiovascular health factors in healthy individuals (Helgerud et al., 2007; Talanian et al., 2007; Hood et al., 2011). Recent research has indicated that short bouts of vigorous intensity exercise may drive the inverse association between physical exercise and cardio-metabolic risk factors (Hay et al., 2012; Carson et al., 2014; Logan et al., 2016). Therefore, higher intensity activities performed in short repeated bouts, interspersed with periods of recovery, may be an achievable and more enjoyable alternative to high-volume continuous exercise (Logan et al., 2014, 2016). Several studies, including a meta-analytic study, reported that participants prefer and enjoy interval exercise just as much, if not more, than continuous exercise (Stork et al., 2017; Oliveira et al., 2018). HIIT is widely accepted as an exercise mode effective in improving aerobic fitness, body composition, insulin sensitivity, blood lipid profile, blood pressure, and cardiovascular function (Burgomaster et al., 2008; Babraj et al., 2009; Kemi and Wisloff, 2010). While research studying the efficacy of HIIT on the health-related outcomes of non-exercisers, especially older people, has been limited, initial studies indicate similar or superior benefits using HIIT when compared to MCE (Logan et al., 2014; Costigan et al., 2015).

Exergames, a portmanteau of exercise and gaming, “aims to balance physical activity and gameplay to provide the user with a fun and engaging gaming experience while ensuring that physical player activity leads to significant levels of energy expenditure (Gerling and Mandryk, 2014).” A comprehensive systematic review provides an extensive overview of existing studies with exergames and their effects on older adults’ level of physical activity (Kappen et al., 2018). The findings support the usage of exergames in older adults, as shown by increased motivation and engagement of older people in general physical activity. Further systematic reviews have summarized evidence supporting the effectiveness of exergames to treat age-related chronic disease in terms of physical, psychological, and cognitive rehabilitative outcomes (Zeng et al., 2017; van Santen et al., 2018). However, research focusing on exergame effects on aerobic capacity is largely unavailable. In the current context it is assumed that exergames are merely able to elicit light intensity energy expenditure when employed in community-dwelling older people (Taylor et al., 2018). However, exergaming has also been described as an acceptable, safe (Valenzuela et al., 2018) and innovative way to design enjoyable HIIT (Moholdt et al., 2017). So far, rather few studies and game developers have specifically considered aerobic training in general (Skjaeret et al., 2016) and HIIT specifically for senior target populations. Peng et al. (2011) showed that exergames may facilitate light- to moderate-intensity physical activity. However, the effects of different exergame training protocols or the possibility to perform high-intensity training through exergames, have not yet been systematically investigated.

This pilot trial aimed to develop and refine a theory-based exergame-driven HIIT training protocol for untrained older adults and to estimate potential efficacy. The main objectives were [1] determining feasibility by evaluating user acceptance and usability of a stepping-based exergame protocol and [2] assessing preliminary effects on cardiovascular fitness in untrained community dwelling older participants.

## Materials and Methods

### Study Design

**FIGURE 1:**
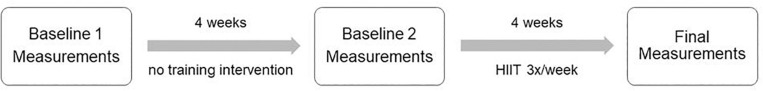
Study flowchart.

Each trainee was involved in the study for a period of 8 weeks using a quasi-experimental single group repeated measures design. There were two study phases, a 4 weeks baseline phase and a 4 weeks Intervention phase, with measurements taken at the start of each phase and one measurement at the end of the second phase (Figure 1). In this way the older adults acted as their own controls. This approach controls for inter-subject variability (Willerslev-Olsen et al., 2015), which would complicate the comparison of data from different subjects, given an expected diversity of fitness levels. This design also allowed determining whether any effect of the intervention can be explained by simple test–retest variability and whether effects on estimates of the group means and group-specific variability (Moore et al., 2011) would be observable in the measured variables during a 4-week control period similar to that of the 4-week intervention period. The study was carried out from July to November 2017, and the study protocol was approved by the Research Ethics Committee of ETH Zürich, Switzerland (Protocol No. EK 2017-N-26) and conforms to the Declaration of Helsinki. All participants were fully informed prior to participation and signed informed consent. All trainings and measurements were performed at ETH Zürich, Switzerland. We adhered to the CONSORT 2010 complement Transparent reporting of evaluations with non-randomized designs (TREND-2004) statement guidelines (Des Jarlais et al., 2004) for the reporting of our quasi experimental feasibility trial.

### Participants

The study was designed for independently living seniors from Switzerland. Subjects had to be naïve regarding exergames. Elderly people who were interested to participate were considered eligible if they were: untrained or non-regular exercisers (by self-report); having good health (by self-report); scoring 26–30 points on the MoCA; understanding written and verbal information in German. Criteria for exclusion were: subjects with acute or unstable chronic diseases (e.g., recent cardiac infarction, uncontrolled diabetes or uncontrolled hypertension) and those with rapidly progressing or terminal illnesses; Alzheimer disease patients, people suffering from dementia, or individuals with a recent head injury; participation in regular (>2x/week) endurance exercise training (currently or in the last year); participation in a conflicting exercise study or answering “yes” on one or more questions on the ParQ (Cardinal et al., 1996): in case one or more questions of the ParQ were answered “yes,” the participant was advised to consult a physician for medical clearance before entering the study.

### Sample Size Considerations

The aim was to recruit up to 16 untrained, healthy by self-report, community dwelling older participants from the greater area of Zürich via local newspaper advertisements, bulletins, advertisement boards at local shops and/or through personal contact via staff members of the study. A relative small sample was deemed appropriate for the main objective of testing the feasibility of our new HIIT training (Moore et al., 2011). Furthermore, to inform the design of a larger more comprehensive study based on confidence interval (CI) width for mean response(s) acceptable precision may be expected from a *N* between 10 to 15 trainees (Moore et al., 2011).

### HIIT Intervention

**FIGURE 2:**
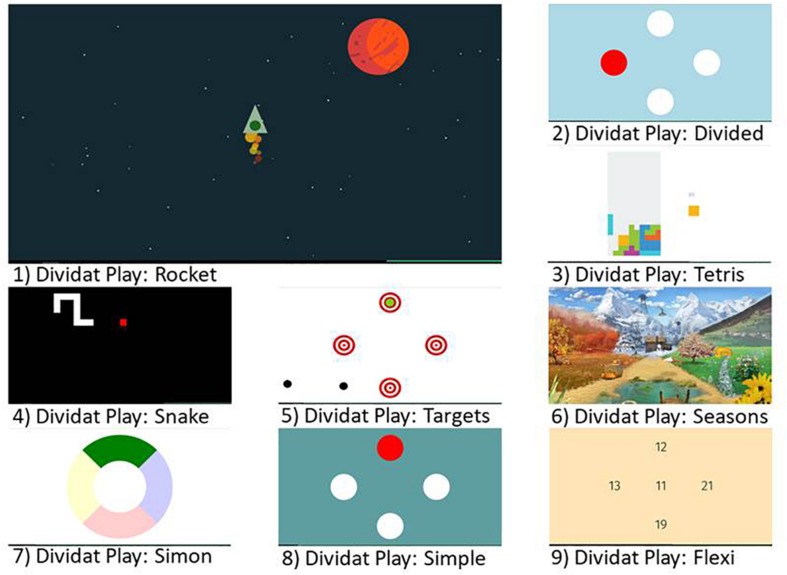
Screenshots of the used exergames.

Based on theoretical considerations (Wuest et al., 2014b) combined with exercise recommendations for older adults (de Souto Barreto et al., 2016) and knowledge from an effectively implemented conventional type HIIT training in older adults (Robinson et al., 2017), a program that consisted of an exergame-based HIIT performed while standing on a pressure sensitive platform (Dividat SENSO, Schindellegi, Switzerland, 93/42/EWG certified) was designed as follows: short intervals (one up to 2 min) of higher intensity exertion [Rocket-game (game one); at 70–90% of HRmax] were alternated with active rest periods (2 min down to 1 min at 50–70% of HRmax) for a total of up to 25 min. The intervention was performed individually three times per week (to be in line with exercise recommendations related to frequency of training) for a period of 4 weeks with a total of 12 sessions. Each training session was partitioned into three parts: (i) 5 min warming-up on a cycle ergometer (Ergoline Ergoselect 50, Bitz, Germany) at 50–70% of HRmax, followed by (ii) up to 25 min of HIIT with the Rocket-game, interspaced with different exergames to be played in the low-intensity phases (games two to nine; Figure 2) on the pressure sensitive platform. The sessions were finished with (iii) a cool-down of 5 min at 50–70% of HRmax on the cycle ergometer. Participants were expected to complete 12 training sessions while being monitored by a trainer, who systematically observed and supervised participants throughout the training. The training intensity was individually tailored to each participant and progressed according to a training protocol based on guidelines published by the American College of Sports Medicine (Chodzko-Zajko et al., 2009) and aimed at increasing endurance capacity. Table 2 includes an exemplified HIIT protocol for the exergame training program. The complete intervention was performed in a laboratory exercise room of the ETH Zürich, location Hönggerberg, Switzerland. To enable replication of the intervention the Template for Intervention Description and Replication (TIDIER) (Hoffmann et al., 2014) was used.

To determine the individual training load, a graded maximal exercise test on a cycle ergometer, as used by Broman et al. (2006), was performed to evaluate maximum workload (in Watt, W) and HRmax. To account for the discrepancy between HRmax evaluated on a cycle ergometer vs. a treadmill, target HRmax was calculated as HRmax + 10% (Mitchell et al., 2010). Participants performed a warm-up of 5 min at 40 W followed by an incremental maximal exercise test. Exercise load started at 40 W and was raised each minute by 20 W until exhaustion. The pedal rate was set to 60 rpm (revolutions per minute). Heart rate was continuously monitored with a heart rate sensor (Polar 10, Kempele, Finland). Criteria for exhaustion were (i) reaching a rate of perceived exertion greater than 17 (participants received a standard instruction on the 6–20 rating of perceived exertion scale and were asked to focus on their ‘whole body’ perception of exertion) (Reed and Pipe, 2015), (ii) a maximal heart rate that fails to increase with higher workload, (iii) a pedal rate that falls under 60 rpm for more than 1 min or (iv) the participants’ request to stop.

#### Exergames

**FIGURE 3:**
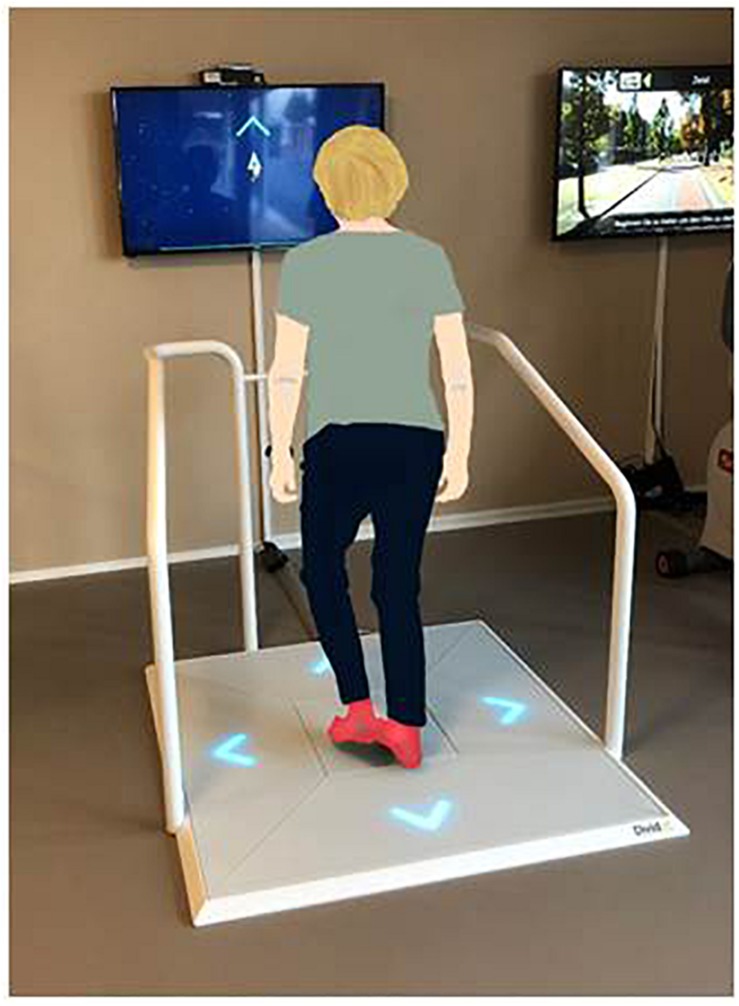
The Senso exercise system with the Rocket game displayed on screen.

A set of nine exergames (Figure 2; Dividat, Schindellegi, Switzerland), was used for the training intervention. The main Rocket-exergame was purpose designed to achieve the high-intensity intervals. Participants had to pilot a rocket through the universe and keep it above a threshold line through performance of high-frequency steps on the pressure sensitive plate (Figure 3). Pace and number of steps per second were modulated according to current heart rate and perceived exertion [Borg Scale 6–20, which tries to match the number on the scale to the person’s heart rate (Borg, 1982)]. The faster the performed steps on the SENSO, the more the rocket accelerated. The additional eight exergames (‘Divided,’ ‘Flexi,’ ‘Seasons,’ ‘Simon,’ ‘Simple,’ ‘Snake,’ ‘Targets,’ and ‘Tetris’) were used during the active rest periods between the high intensity bouts. These games target EF of the brain (Eggenberger et al., 2015a, 2016) that are related to physical functions; e.g., gait variability (Eggenberger et al., 2015b; Schattin et al., 2016). These eight additional games were deployed to make the training more enjoyable for participants, and it was assumed that a low intensity of around 50–70% of HRmax would be attained during these active rest periods.

Participants were encouraged to “think aloud,” a procedure often used in video game development studies (Hwang and Salvendy, 2010; Pirker and Gütl, 2015; May, 2019), while operating the software and playing the exergames (Wuest et al., 2014a; Eccles and Arsal, 2017). Any expressed comments from the participants related to their demands were documented by the supervising person in writing to identify both problem areas and what people like. A heuristic tailored to video games and with a focus on the usability of the exergames by the older adults (Desurvire et al., 2004; Pinelle et al., 2008; May, 2019) was used assuming that this video game heuristic would have more specificity (May, 2019).

The aim was to assess strengths and weaknesses of the exergame-based training program online in a realistic setting (McBain et al., 2018). Based on participants’ feedback, information on possible exergame adaptations could be gained.

### Data Collection

Endpoints were evaluated during three separate test sessions at intervals of approximately 4 weeks (Figure 1). The first test session was performed 4 weeks before onset of HITT, and a second test procedure was performed before the first training session. The last measurement session took place at least 2 days following the last training at the end of week 8. All three test sessions included the same measurements conducted in the same order and, where possible, at the same time of day. Data collection was performed by the same senior physiotherapist (SR) supervising the HIIT intervention.

### Outcomes

#### Primary Outcomes

The criteria for success were based on the feasibility objective and focused on adherence and attrition to the intervention, acceptability and usefulness questionnaires next to making comparisons between participants’ physical activity levels pre and post-intervention against values of training intensity, frequency, duration and exertion for similar types of training (Reed and Pipe, 2015). Adherence to the intervention was calculated as the number of completed training sessions in relation to the amount of planned training sessions. An adherence rate of 80% was deemed acceptable and for attrition, the number of participants lost during the trial, a 10% threshold was defined *a priori* (Nyman and Victor, 2012; Skjaeret et al., 2016).

Acceptability and perceived usefulness of the training technology were evaluated with the TAM, which consists of a 15 item questionnaire evaluating participants’ perceived usefulness, ease of use, attitude toward using and behavioral intention to use (Davis, 1989; Knols et al., 2017) of the technology. Answers were recorded on a seven-point Likert scale ranging from ‘strongly disagree’ (rated as 1) to ‘strongly agree’ (rated as 7). Usability was assessed with the German version of the reliable SUS (Brooke, 2013). This questionnaire consists of 10 items with five response options: from ‘strongly disagree’ (rated as 0) to ‘strongly agree’ (rated as 4). Participants’ general statements while answering these questionnaires were used together with expressions from training experience for a better understanding of the intervention and participant’s beliefs about the intervention.

The overall enjoyment of exergaming was evaluated on a scale ranging from ‘not enjoyable’ to ‘highly enjoyable’ (Sell et al., 2008).

#### Secondary Outcomes

Secondary outcomes assessed the effects of the HIIT program on cardiovascular fitness and consisted of HRrest, HRV, HRmax, and maximum workload (W, in watt) and $\dot{\text{V}}\,{\text{O}}_{2}\text{max}$ evaluated through graded maximal exercise testing.

Participants arrived at the measurement site and rested for 10 min in a sitting position (no talking or moving) before HRrest [beats per min (bpm)] was measured with a heart rate sensor (Polar 10, Kempele, Finland). HRrest represents the minimal value of heart rate measured during the last minute of the resting period (Racil et al., 2016) and is considered an important prognostic marker for primary and secondary prevention (Nauman, 2012).

**FIGURE 4:**
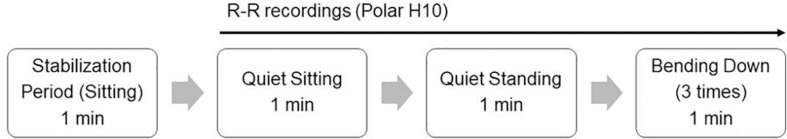
HRV measurement protocol.

Heart rate variability, measured according a protocol by Hautala et al. (2016), sampled beat-to-beat (R-R intervals) variations with a timing accuracy of 1 ms after a 1-min stabilization period in a sitting position. After the stabilization period, R-R interval data was recorded during sitting, standing and bending down (Figure 4). Mean R-R intervals (mean RRi, ms), as well as HRmean (bpm), standard deviation of R-R intervals (mean SD1, ms) and the root-mean-square difference of successive normal R-R intervals (mean rMSSD, ms) were issued separately for the three different tasks. SD1 measures the magnitude of vagally mediated beat-to-beat variability in the data as standard deviation of the data points from the identity line (Hautala et al., 2016). rMSSD has been proven a robust measure of cardiac vagal activity and has been used specifically in short-term HRV (1 min) assessment (Flatt and Esco, 2016; Hautala et al., 2016). From previous research we know that the type and duration of physical activity interacts with HRV of sedentary individuals (Tulppo et al., 2003).

HRmax and maximum workload (in Watt, W) were determined according to a graded maximal exercise test on a cycle ergometer as described above. The protocol of Uth et al. (2004) was used to determine a proxy measure of $\dot{\text{V}}\,{\text{O}}_{2}\text{max}$ based on the results of the graded maximal exercise test. $\dot{\text{V}}\,{\text{O}}_{2}\text{max}$ depends on exercise intensity, duration, frequency, and program length in sedentary older adults (Huang et al., 2016).

### Statistical Analysis

Only those individuals who adhered to the training protocol and did not drop out counted toward the results (per protocol analysis). IBM SPSS Version 24.0 software (SPSS, Inc., Chicago, IL, United States) was used for data analysis. The study population’s clinical characteristics and the primary exergaming related feasibility outcomes were calculated using descriptive statistics. An average percentage of the different feasibility variables matching their target range as well as the overall range of the feasibility variables were calculated.

Training data (HRrest, HRV, Workload, HRmax, $\dot{\text{V}}\,{\text{O}}_{2}\text{max}$) were analyzed using point and interval estimate values from the first and second phase of the intervention. Normality of the data was evaluated using the Shapiro–Wilk test. Depending on normality or non-normality of the data, ANOVA and accordingly the non-parametric repeated measures comparison (Friedman test) was used to compare the two pre-training and the post-training results. Bonferroni-corrected *post hoc* analyses were carried out if ANOVA was significant and the Dunn-Bonferroni *post hoc* tests applied to significant Friedman tests. For effect estimation interpretation, confidence intervals were used (Sim and Reid, 1999; Stratford, 2010; Lee et al., 2014). The level of statistical significance was set to *p* < 0.05.

Effect sizes were calculated for within-group differences and expressed as *r* = z/√N (for non-normality of data), where z is the approximation of the observed difference in terms of the standard normal distribution and N is the total number of samples (*r* = 0.1, small effect; *r* = 0.3, medium effect; and *r* = 0.5, large effect) (Fritz et al., 2012). Cohen’s *f* = (ηp/21-ηp)2 was reported in the context of a *F*-test for ANOVA (*f* = 0.1, small effect, *f* = 0.25, medium effect, *f* = 0.4, large effect) (Cohen, 1988).

## Results

### Primary Feasibility Outcomes

Twenty persons initially approached (15 women and 5 men), were interested in participating and screened for eligibility. Eight individuals were excluded before the first baseline measurement (40%): one person due to chronic illness, two because of lack of time, two because of being more physically active than allowed, and three due to personal reasons. Twelve participants signed the consent form and were included in this study (2 men, 10 women), aged 72.3 (*SD:* 4.44) years. These remaining participants had normal or corrected to normal vision. Table 1 provides demographic and clinical characteristics of the sample.

**TABLE 1:** Demographics and clinical characteristics of participants (*n* = 12).

	**Mean**	***SD***	**Median**	**Range**
Age (years)	72.33	4.44	72.50	65–79
Height (m)	1.65	0.07	1.66	1.58–1.83
Weight (kg)	69.29	9.68	71.75	52–83
BMI	25.34	2.95	25.55	20.3–29.2
MoCA	–	–	28	26–30

**Computer-use/game-use**	***n***	**%**	***n***	**%**

Daily	12	100	2	16.7
Weekly	–	–	3	25
Monthly	–	–	1	8.3
Seldom/never	–	–	6	50

**Education level**	***n***	**%**		

Secondary school	1	8.3		
Vocational education	9	75		
Higher professional education	–	–		
College/University	2	16.7		

*SD, standard deviation.*

**TABLE 2:** Scheduled training protocol.

**Training period**	**Target times and intensities**	**Remark**
**Week 1** (Trainings 1–3)	**High-intensity interval**: 4 min × 1 min up to 9 min × 1 min at 70–80% of HRmax + 10%, Borg 12–14	If participant was already working at ∼80% level of HRmax + 10% at the end of week 1, keep continuing at ∼90% level of HRmax + 10%
	**Active recovery interval:** up to 8 min × 2 min at 50–70% of HRmax + 10%	
**Week 2** (Trainings 4–6)	**High-intensity interval**: 3 min × 2 min up to 6 min × 2 min at 70–80% of HRmax + 10%, Borg 12–14	If participant was already working at ∼80% level of HRmax + 10% at the end of week 2, keep continuing at ∼90% level of HRmax + 10%
	**Active recovery interval:** up to 5 min × 2 min at 50–70% of HRmax + 10%	
**Week 3** (Trainings 7–9)	**High-intensity interval**: 3 min × 2 min up to 6 min × 2 min at 80–90% of HRmax + 10%, Borg 15–17	
	**Active recovery interval:** up to 5 min × 2 min at 50–70% of HRmax + 10%	
**Week 4** (Trainings 10–12)	**High-intensity interval**: 5 min × 2 min up to 8 min × 2 min, at 80–90% of HRmax + 10%, Borg 15–17	
	**Active recovery interval:** up to 7 min × 1 min at 50–70% of HRmax + 10%	

*HRmax, maximum heart rate.*

None of the participants experienced any adverse events during the intervention. One participant dropped out after the first baseline measurement due to acute illness unrelated to the study, resulting in an attrition rate of 8%. Adherence to the intervention resulted in 91% compliance (attendance rate: 120/132 sessions; range: 82–100%).

Total exercise time ranged from 19 to 35 min with a mean of 30.8 (*SD*: 3.6) min. Total high-intensity exercise time showed consistency with the target exercise time in 98% of trainings, while the total active recovery time presented with 99% accuracy.

**FIGURE 5:**
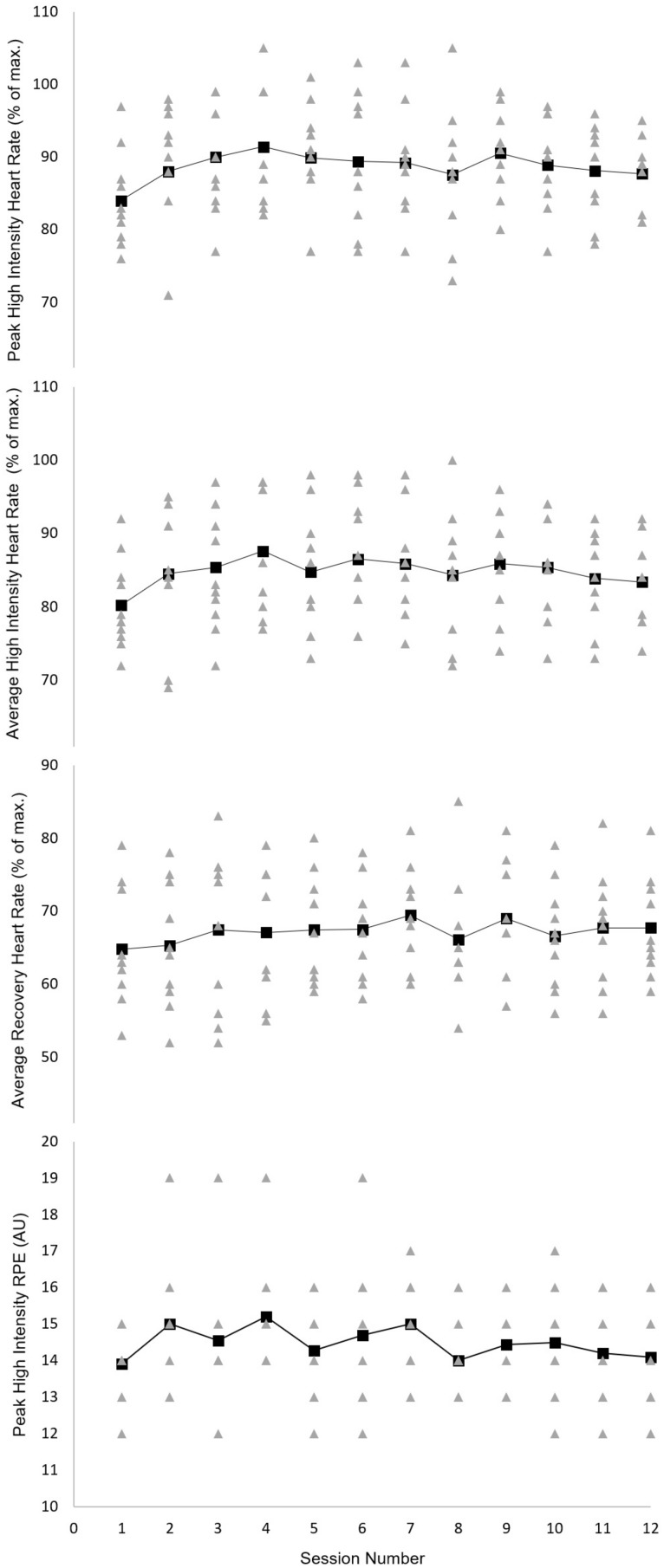
Group mean (large black squares) and individual (small gray triangles) heart rates (high intensity interval peak, mean and recovery interval mean) and peak ratings of perceived exertion (RPE) across the 4-week exergaming intervention period (session numbers 1–12). AU, arbitrary unit; RPE, rating of perceived exertion.

Peak percentage of HRmax ranged from 71 to 105%. The average performed percentage of HRmax during the high-intensity intervals was consistent with the proposed schedule in 86% of cases (range: 69–100%), with an average of 14% of intervals below target intensity. In contrast, average performed percentage of HRmax during recovery intervals showed a 63.5% accuracy (range: 52–85%), with an average of 36.5% of intervals above target intensity. Rating of perceived exertion during the high-intensity intervals ranged from 12 to 19 with a median of 14-and-a-half (Figure 5).

Table 3 presents the mean and standard deviation values for the TAM questionnaire items that were evaluated post-intervention. In general, the ‘Behavioral Intention to Use’ and the ‘Attitude Toward Using’ showed high levels of motivation to use the exergames on a regular basis. Moreover, the videogames were perceived as useful to increase training performance.

**TABLE 3:** Evaluation of the Technology Acceptance Model Questionnaire (TAM).

	**Mean**	***SD***	**Range**
Perceived Ease of Use	6.7	0.6	5–7
Perceived Usefulness	6.0	1.1	3–7
Attitude Toward Using	6.2	0.9	4–7
Behavioral Intention to Use	5.8	1.3	2–7

*SD, standard deviation.*

Participants rated the usability of the exergames with an overall score of 93.5 (*SD*: 5.52) as ‘excellent’ (Brooke, 2013) and reported to enjoy the exergaming with 4.5/5 (*SD:* 0.5) points.

#### Qualitative Findings

TAM, SUS, and training data revealed that participants found the step plate and exergames to be clear and understandable, as emphasized in the following quotes:

“I feel that using the exergames on my own would be easy for me. At the beginning it was challenging, but with time I realized that I became skillful at using the different options and possibilities of the system. Now I’m confident and like the idea of using the exergames on my own in the future.”

Participant 11, 75 years

“I didn’t need to learn a lot of things before I could get going with this system. The support of a technical person would not be needed to be able to use this system, even at my age. There’s no unnecessary complexity. You can feel that the games were designed to be played by older people.”

Participant 12, 79 years

Most of the participants stated that the various functions in the system were well-integrated, but found some inconsistencies and would have wanted the Rocket-game to be more engaging:

“I felt very confident using the system and most of the games were excellent and motivated me to get better and better. There were some minor inconsistencies with the step plate or the application. Fortunately, these problems could be solved easily. Adding music to the game-play would even make it more enjoyable.”

Participant 2, 71 years

“The Rocket-game could be more challenging by integrating obstacles in the virtual space environment, that have to be avoided through direction changes of the rocket. I would also have liked the possibility to collect or loose points and more visual feedback. This would have made me try even harder at the game.”

Participant 6, 69 years

Finally, participants liked the idea of combining training with game-playing and confirmed that it was encouraging and helped them to increase their training performance as illustrated in the following quote:

“At the beginning I was skeptical about the exergaming, but as I carried on, I realized that it helped me forget about the exertion. The exergames made me focus on the fun side of gaming rather than the exercise. Assuming that I have access to the step plate and exergames, I intend to use them regularly in the future.”

Participant 8, 75 years

### Secondary Outcomes

Table 4 summarizes the secondary outcome variables with corresponding confidence intervals.

**TABLE 4:** Exercise capacity data at three different time points.

	**Mean (*SD*)**		
**Variables**	**Baseline 1**	**Baseline 2**	**Final test**
**HRrest** (bpm)	66.7 (15.7)	72.6 (16.6)	68.3 (14.5)
95% CI	[57.8, 75.6]	[62.8, 82.4]	[59.7, 76.9]
**HRV data**			
Sitting meanRRi (ms)	934.3 (220.2)	861.3 (204.4)	850.0 (175.5)
95% CI	[809.7, 1058.8]	[740.5, 982.1]	[746.3, 953.7]
Sitting meanSD1 (ms)	36.5 (26.2)	30.4 (25.7)	37.9 (25.3)
95% CI	[21.7, 51.3]	[15.2, 45.6]	[22.9, 52.9]
Sitting rMSSD (ms)	33.3 (35.7)	28.6 (35.2)	30.2 (31.8)
95% CI	[13.1, 53.5]	[7.8, 49.4]	[11.4, 48.9]
Standing meanRRi (ms)	855.4 (228.8)	797.4 (204.6)	777.9 (201.1)
95% CI	[725.9, 984.9]	[676.5, 918.3]	[659.1, 896.7]
Standing meanSD1 (ms)	35.8 (25.6)	31.6 (30.0)	35.6 (29.7)
95% CI	[21.3, 50.3]	[13.9, 49.3]	[18.1, 53.2]
Standing rMSSD (ms)	28.9 (35.3)	27.6 (37.3)	27.7 (31.2)
95% CI	[8.9, 48.9]	[5.6, 49.6]	[9.3, 46.1]
Bending meanRRi (ms)	824.7 (200.6)	765.3 (190.5)	769.8 (171.0)
95% CI	[711.2, 938.2]	[652.7, 877.9]	[668.7, 870.9]
Bending meanSD1 (ms)	53.0 (38.3)	59.9 (45.9)	46.8 (39.5)
95% CI	[31.3, 74.7]	[32.8, 87.0]	[23.5, 70.1]
Bending rMSSD (ms)	47.4 (57.8)	54.1 (64.4)	44.1 (52.2)
95% CI	[14.7, 80.1]	[16.0, 92.2]	[13.3, 74.9]
**Incremental test parameters**			
Workload max (W)	120.0 (21.9)	118.2 (30.3)	136.4 (23.4)
95% CI	[107.6, 132.4]	[100.3, 136.1]	[122.6, 150.2]
HRmax (bpm)	134.9 (21.2)	135.2 (20.5)	138.3 (20.6)
95% CI	[122.9, 146.9]	[123.1, 147.3]	[126.1, 150.5]
**$\dot{\text{V}}\,{\text{O}}_{2}\text{max}$** (ml/kg^∗^min)	31.8 (5.2)	29.2 (4.1)	31.8 (5.3)
95% CI	[28.9, 34.7]	[26.8, 31.6]	[28.7, 34.9]

*Bpm, beats per minute; CI, confidence interval, HRmax, maximum heart rate; HRrest, heart rate at rest; HRV, heart rate variability, meanRRi, mean R-R interval; meanSD1, standard deviation of R-R intervals; ms, millisecond; rMSSD, root-mean-square difference of successive normal R-R intervals; SD, standard deviation; $\dot{\text{V}}\,{\text{O}}_{2}\text{max}$, maximal oxygen uptake (ml/kg^∗^min); W, Watt.*

Data were approximately normally distributed except for maximal workload at 4 weeks and some of the HRV measurements. A repeated measures analysis of variance (ANOVA) with sphericity assumed determined that mean HRVsitting meanRRi and HRVbending meanRRi differed significantly between time points [*F*(2,20) = 4.843, *p* = 0.019^∗^ and *F*(2,20) = 5.307, *p* = 0.014^∗^, respectively]. No significance was detected in the Bonferroni-corrected *post hoc* tests for these variables.

The ANOVA for mean HRrest found a statistically significant difference between time points [*F*(2,20) = 6.621, *p* = 0.006^∗^]. *Post hoc* tests showed an increase in HRrest during the 4 weeks without training [66.7 (*SD*: 15.7) bpm vs. 72.6 (*SD*: 16.6) bpm, respectively], which was statistically significant (*p* = 0.025^∗^). However, post-training HRrest reduced to 68.3 (*SD*: 14.5), which was not statistically significant, but showed a trend toward significance (*p* = 0.070).

Mean $\dot{\text{V}}\,{\text{O}}_{2}\text{max}$ differed statistically significant between time points [*F*(2,20) = 7.048, *p* < 0.005^∗^]. *Post hoc* tests revealed a decline in mean $\dot{\text{V}}\,{\text{O}}_{2}\text{max}$ from measurement one to measurement two [31.8 (*SD*: 5.2) ml/kg^∗^min vs. 29.3 (*SD:* 4.1) ml/kg^∗^min, respectively], which was statistically significant (*p* = 0.030^∗^) and a significant increase in mean $\dot{\text{V}}\,{\text{O}}_{2}\text{max}$ from measurement two to measurement three [29.2 (*SD*: 4.1) ml/kg/min vs. 31.8 (*SD*: 5.3) ml/kg/min, respectively, *p* = 0.033^∗^].

Table 5 shows the results of the Friedmann tests used to evaluate the relationship between the three measurement time points and the non-normally distributed data.

**TABLE 5:** Comparison of pre-/post-training results (Friedman tests).

	**Friedman test**			***Post hoc*: Dunn-Bonferroni**		
	**Chi^2^(2)**	***p*-Value**	***n***	***Z***	***p*-Calue**	***r* = | z/√n|**
sitting meanSD1	0.000	1.000	11	–	–	–
sitting rMSSD	0.182	0.913	11	–	–	–
standing meanRRi	2.182	0.336	11	–	–	–
standing meanSD1	3.818	0.148	11	–	–	–
standing rMSSD	0.182	0.913	11	–	–	–
bending meanSD1	5.636	0.060	11	–	–	–
bending rMSSD	2.364	0.307	11	–	–	–
Workload max	13.688	0.001^∗^	11			
	Baseline 1 vs. final test			−2.558	0.032^∗^	|0.77|
	Baseline 2 vs. final test			−2.878	0.012^∗^	|0.87|

*meanRRi, mean R-R interval; meanSD1; standard deviation of R-R intervals; rMSSD, root-mean-square difference of successive normal R-R intervals. All non-normally distributed data were analyzed with the non-parametric repeated measures comparison (Friedman test). Effect size: *r* = Pearson correlation coefficient (*r* = 0.1, small effect; *r* = 0.3, medium effect; and *r* = 0.5, large effect). ^∗^Significance level was established at *p* < 0.05.*

## Discussion

This formative trial followed the objective of determining the feasibility of an exergaming approach by evaluating user acceptance and usability of a newly developed HIIT exergame protocol next to assessing preliminary effects on cardiovascular fitness in untrained healthy older participants. To the best of our knowledge, this feasibility study is the first to explore the use of exergames as a high-intensity interval endurance training method in an untrained elderly population. Our findings reveal a low attrition rate and high scores for each of the four TAM items, resulting in a high level of acceptance, that likely accounts for the high adherence rate to the intervention. This high adherence is in line with findings from a systematic review showing that higher adherence rates to technology-based interventions may be expected from older adult trainees who seem to rather well-accept technology-based exercise opportunities (Valenzuela et al., 2018). Our formative feasibility study demonstrates the feasibility of achieving both the targeted exercise intensity and the interval character of the intervention through exergaming. In the context of developing new and complex interventions obtaining information on whether the intervention can be used as intended by the target population is of great importance (Moore et al., 2011). This information allows modifications to the training protocol and informs the design of larger, more comprehensive studies. Based on these first results we are confident that the exergame was used as intended and can now be tested in a clinical trial following some small amendments to the exergame while continuing to closely monitor the participants (Thabane et al., 2010). The proposed amendments to the Rocket-game are based on some comments from the trainees, expressed while performing their training. Adding music to the game-play was mentioned several times. Furthermore, making the Rocket game more challenging could be reached by integrating obstacles in the virtual space environment; e.g., meteors, rocks, etc., that have to be avoided through direction changes of the rocket. Participants also requested more visual and auditive feedback while playing the game and the possibility to collect or lose points.

None of the participants experienced any adverse events such as dizziness, pain, or cardiovascular issues during the trainings. The results are in line with another cycle-based HIIT exergame tested on eight young males that were used to playing computer games for more than 5 h/week (Moholdt et al., 2017). This exergame was, however, not tested with a longitudinal intervention approach. The results of this within-subject study design revealed that heart rate (HR), activity duration, caloric expenditure and subject-rated exertion and enjoyment were all superior when the participants played the exergame as compared to walking outside (Moholdt et al., 2017).

Compared with a median rate of close to 90% for adherence in exercise and rehabilitation delivered through exergames in older adults (Skjaeret et al., 2016; Valenzuela et al., 2018), our study achieved a similarly high level. Our study’s high adherence to the intervention rate of 91% can be attributed to the short length of the training period, the engaging and divers selection of exergames, the constant presence of a trainer, and the less time consuming HIIT protocol compared to MCE (Picorelli et al., 2014; Stork et al., 2017). The TAM questionnaire’s ‘Behavioral Intention to Use’ as well as the high value in ‘Attitude Toward Using’ showed that participants were motivated to use the exergames on a regular basis. Moreover, the ‘Perceived Usefulness’ results demonstrated that the videogames were perceived as useful means to increase training performance, productivity and effectiveness. The high acceptance rate found in the current study further agrees with previous studies using specifically designed and developed exergames in older adults (Wuest et al., 2014a; Konstantinidis et al., 2016). Overall, participants rated the usability of the exergames as ‘excellent,’ which is much higher than other studies encountered (Vaziri et al., 2016). Slight disagreement was apparent on the integration of the various functions and the amount of inconsistency in the system. The overall enjoyment of exergaming was rated as high (4.5/5), which is an important measure according to Hochsmann et al. (2016), who found a significant correlation between energy expenditure and enjoyment of exergaming. Enjoyment, furthermore, is predictive of physical activity participation and expected enjoyment from physical activities can increase exercise intentions (Mullen et al., 2011).

A secondary aim of this study was to acquire information on the estimate of the treatment effect (efficacy) of exergame-driven HIIT related to cardiovascular parameters and, thus, provide a basis for calculating sample sizes needed for randomized control trials. Some main findings for the secondary outcomes were a trend toward significant change for the mean HRrest and a significant difference in mean $\dot{\text{V}}\,{\text{O}}_{2}\text{max}$ in the intervention phase. To estimate the clinical importance related to these findings we compared the resulting confidence intervals from our study (Sim and Reid, 1999) with observed change values reported in two meta-analytic studies (Huang et al., 2016; Reimers et al., 2018). Females and males that perform regular endurance training show a mean relative change of −4.7 ± 0.7% in resting heart rate (Reimers et al., 2018). The relative change observed in our trial amounts to −5.9%, which compares favorably when we consider the rather short intervention period of 4 weeks only. For the mean $\dot{\text{V}}\,{\text{O}}_{2}\text{max}$ values a change that might be expected due to trainings that range in length between 8 and 52 weeks would be 3.78 ml/kg^∗^min (Huang et al., 2016). The value we observed following 4 weeks of HIIT amounted to 2.6 ml/kg^∗^min based on a conservative approach by using the 95% confidence interval. Lee et al. (2014) suggested that for pilot trials 85 and 75% confidence intervals should be applied and related to the clinical meaningful difference for the estimation of needed sample sizes in future confirmatory trials. It seems clear that our secondary outcome values should be interpreted with prudence; however, the sizes of the estimates seem rather encouraging and warrant to proceed to a main trial.

The most prominent finding for the secondary outcomes was the highly significant differences in maximum power output (W) achieved during the incremental exercise test. While HRmax did not significantly change, the maximal workload increased significantly from baseline one and two to the final measurement with ESs as high as *r* = 0.77 and *r* = 0.87 respectively. This suggests that after 4 weeks of HIIT through exergames, participants were able to reach a significantly higher workload at a slightly but not significantly increased HRmax and unchanged RPE. According to MacInnis and Gibala (2016), this improvement may be attributed to an increased mitochondrial content and capillary density as well as improved cardiac output. Whether such effects take place when our program is applied in training programs of extended length should be investigated in future studies exploring the underlying mechanisms of exergame-based HIIT training. Regarding the effect on exercise capacity, Milanovic et al. (2015) pointed out that interventions of longer duration (13 weeks or more) revealed greater beneficial improvements in $\dot{\text{V}}\,{\text{O}}_{2}\text{max}$ compared to shorter HIIT interventions.

With exergaming, moderate-to-vigorous intensity exercises, as based on guidelines proposed by ACSM^TM^ or WHO for health and fitness benefits, are achievable in persons with neurological disabilities. These activities may, at the same time, be perceived as more engaging and enjoyable then conventional training. For persons with neurological disabilities, exergaming should be viewed by the clinician as “at least as good as” (and likely more enjoyable) than traditional arm-exercise modalities, with equivalent aerobic dose-potency as “traditional” exercise in clinic or home environments (Mat Rosly et al., 2017). To create a holistic and user-centered design, it is recommendable to work with interdisciplinary developer teams of experts from all related fields. In the context of personalized exergames for HIIT and rehabilitation in seniors, the team should consist of older people, therapists, movement and sports scientists, game designers, industrial and interaction designers as well as Human Computer Interaction researchers, who can provide relevant knowledge from their different perspectives (McCaskey et al., 2018).

According to Moholdt et al. (2017), previous studies on the physiological responses to exergaming have found that such game-play elicited light-to-moderate intensity activity. In contrast, our study examined the feasibility of achieving a high exercise intensity of 70–90% of HRmax or more in older adults. The exercise intensity during the high-intensity bouts was mostly high, with an average of 85% of HRmax. 86% of the high-intensity intervals met the proposed average target range, resulting in 14% of intervals below target range. We observed no tendency of higher HR during the second half of the trial, although target intensity was increased. A likely explanation may be found in the higher than anticipated intensity during the first half of the trial and in the increase of the high-intensity interval from 1 to 2 min from week 2 onwards. Participants accomplished the targeted exercise times during 98% of cases. Although HIIT varies broadly according to different studies, our HIIT protocol and the average HIIT time of 30.8 min are consistent with the review of Ito et al. (2016). According to MacInnis and Gibala (2016), more research is needed whether performing longer durations (i.e., higher numbers of high-intensity bouts per session) or greater frequencies of interval training would have beneficial effects on endurance variables. The minimum required ‘total amount of time’ engaged in ‘high intensity work’ of ≥30 min per week suggested by Bacon et al. (2013) was achieved during the second half of our study.

In contrast to the large consistency between targeted and achieved high-intensities, the average performed percentage of HRmax during the recovery intervals only showed 63.5% accuracy with the protocol. 36.5% of recovery periods were completed with a heart rate above the targeted range of HRmax. The eight videogames (games two to nine) used during the active recovery periods have been specifically designed to train EF of the brain. A likely explanation may be found in the high cognitive stress experienced by the participants during the recovery intervals, and thus the higher than expected percentages of HRmax. However, these numbers might as well be related to the partially very high percentages of HRmax reached by some of the participants during the ‘Rocket’ game, resulting in rather high HR values during the recovery periods. Hochsmann et al. (2016) reported significant increases of between 39 and 65% in HRmean during cognitive videogame play.

Our data show that the average percentage of HRmax, when combining all games, was 75.95% (*SD*: 11.64). Therefore, these exergames contributed to a moderate level of exercise intensity.

### Limitations and Future Research

The present study has some limitations that should be mentioned. First, sample size was small and variability in baseline characteristics was high, explaining to some extent the rather low effects of the training. The too short HIIT period of only 4 weeks is another explanation for the partly small effects of the statistical analysis (Milanovic et al., 2015). However, the main aim of our study was feasibility of our training approach and not investigating program effectiveness. A second limitation concerns the gender distribution in our sample, with women outnumbering men by five to one, making it impossible to undertake gender subgroup analysis. From a meta-analysis we know that training differences between sexes should be expected (Reimers et al., 2018). However, the primary focus of this study was on feasibility and acceptance, and older women were much more interested in HIIT via exergames than men, probably representing the main target group for future research and practice. Thirdly, our study may suffer from volunteer bias, hypothesizing that our volunteer participants were more interested in technology than the average elderly. This might explain their positive attitude and acceptance toward the intervention. Another possible limitation of the study is that although we tried to include untrained participants, the threshold was probably set too high and our sample might have been physically more active than the average population. Furthermore, work to rest ratios play an important role on the beneficial effects of HIIT (Knowles et al., 2015). Our study employed a work to recovery interval ratio of 1:2, increasing to 1:1 and ultimately 2:1, assuming that sedentary older persons are likely to better tolerate shorter work to rest ratios. This is further supported by the high adherence to the intervention in our study but may not have had enough impact on the participant’s cardiovascular system and cognitive function.

The use of qualitative data in our analysis bears some limitations as well. While we acknowledge that qualitative methodology has a unique role in understanding the implementation process of an intervention, we also think that the addition of qualitative research methods to pilot studies may help in better understanding the intervention through gaining some understanding about the meaning of the intervention to the participants as well as participants’ beliefs about the intervention (Verhoef et al., 2002; Cooper et al., 2014). The thinking aloud qualitative methodology we used is complementary to interview-based qualitative research methods assessing trainees’ thinking (Eccles and Arsal, 2017). Therefore, future studies could adopt and integrate qualitative research designs using formal interviews to extend the assessment and understanding of this complex Exergame intervention (Pons-Vigues et al., 2019). This approach may lead to an open scientific dialog about different potential ways in which researchers can analyze qualitative data which is believed to be supportive for advancing the field of older adult exergaming experience. Finally, we cannot exclude a measurement bias since the trainer also collected the test-outcomes.

There are several potential ramifications for our HIIT exergame intervention to be used by older adults in the community. However, future trials in dedicated settings should address these ramifications. Future studies should for example be performed in “at home-settings” to determine whether similar rates of attrition, adherence and acceptability are achievable and to rule out they relate to participants doing the HIIT exergame training in a lab-based measurement and training condition under physiotherapist supervision only. This would help to clarify potential participant bias based on social interaction. This would also help to clarify whether older adults can engage at HIIT exergaming on their own without the social interaction of a researcher or physiotherapist. Previous reports have shown that people engage in HIIT training at home in a similar way as when being closely monitored and supervised (Blackwell et al., 2017). There is a call from experts for using innovative technology for the enhancement of physical activities in long term care facilities (de Souto Barreto et al., 2016). Whether the inclusion of exergame devices in dedicated training rooms of such facilities is beneficial, next to conventional types of training equipment, should be assessed as well.

## Conclusion

The results indicate that a HIIT through exergaming is feasible and shows high satisfaction and acceptance in healthy older adults. Feasibility studies provide invaluable results for health workers and information technology developers using innovative systems and might be highly relevant for further research. Our results corroborate previous findings in showing that virtual reality-based approaches for performing endurance training are perceived as usable. Specifically, this study shows that our exergaming-based HIIT had a highly positive effect on maximum power output on an incremental exercise test. Furthermore, the positive trends shown regarding exercise capacity warrant future studies in older adults. In such future studies safety of the intervention during a more extensive intervention period, higher work to recovery ratios as well as a higher-intensity activity should be considered. Those individuals that were compliant to training were all able to progress in intensity and duration of their exercises. In a next development cycle the HIIT exergame should be compared with conventional HIIT training preferably using a randomized controlled design.

## Ethics Statement

This study was carried out from July to November 2017, and the study protocol was approved by the Research Ethics Committee of ETH Zürich, Switzerland (Protocol No. EK 2017-N-26) and conforms to the Declaration of Helsinki. All participants were fully informed prior to participation and signed informed consent.

## Author Contributions

SR and RK developed the research protocol under the lead of EdB. SR conceived the methodology and carried out data acquisition, data analysis, interpretation of data, first version manuscript writing, and manuscript revision. RK participated in methodology conception, data analysis, interpretation, and manuscript writing and revision. PB carried out methodology conception and manuscript revision. EdB supervised progress and helped with methodology conception, final manuscript writing, and critical revision for scientific content. All authors read and approved the final manuscript.

## Conflict of Interest Statement

EdB was a cofounder of Dividat, the spin-off company that developed the video step platform discussed in this manuscript, and is associated with the company as an external advisor. No revenue was paid (or promised to be paid) directly to EdB or his institution over the 36 months prior to submission of the work. The remaining authors declare that the research was conducted in the absence of any commercial or financial relationships that could be construed as a potential conflict of interest.

## Abbreviations

ANOVA: repeated measure analysis of variance
BMI: Body Mass Index
EF: executive function
ES: effect size
HIIT: high-intensity interval training
HR: heart rate
HRmax: maximum heart rate
HRmean: mean heart rate
HRrest: heart rate at rest
HRV: heart rate variability
MCE: moderate-intensity continuous exercise
MoCA: Montreal Cognitive Assessment
ND: normal distribution of the data
ParQ: Physical Activity Readiness Questionnaire
RPE: rating of perceived exertion
rpm: revolutions per minute
SD: standard deviation
SUS: System Usability Scale
TAM: Technology Acceptance Model Questionnaire
$\dot{\text{V}}\,{\text{O}}_{2}\text{max}$: maximal oxygen uptake
